# Inhibition of EphA2 by syndecan-4 in wounded skin regulates clustering of fibroblasts

**DOI:** 10.1093/jmcb/mjae054

**Published:** 2024-12-23

**Authors:** Rebecca Brooks, Xianhui Wei, Mang Leng Lei, Francisca Cisterna Cid, James A Roper, Rosalind C Williamson, Mark D Bass

**Affiliations:** S chool of Biochemistry, University of Bristol, University Walk, Bristol BS8 1TD, UK; School of Biosciences, University of Sheffield, Firth Court, Sheffield S10 2TN, UK; School of Biosciences, University of Sheffield, Firth Court, Sheffield S10 2TN, UK; School of Biosciences, University of Sheffield, Firth Court, Sheffield S10 2TN, UK; S chool of Biochemistry, University of Bristol, University Walk, Bristol BS8 1TD, UK; S chool of Biochemistry, University of Bristol, University Walk, Bristol BS8 1TD, UK; S chool of Biochemistry, University of Bristol, University Walk, Bristol BS8 1TD, UK; School of Biosciences, University of Sheffield, Firth Court, Sheffield S10 2TN, UK

**Keywords:** syndecan-4, EphA2, cell migration, wound healing, receptor crosstalk

## Abstract

Upon injury, fibroblasts in the surrounding tissue become activated, migrating into the wound in a controlled manner. Once they arrive, they contract the wound and remodel the stroma. While certain cell surface receptors promote fibroblast migration, others cause repulsion between fibroblasts upon contact, seemingly opposing their clustering within the wound bed. Eph receptor–ephrin interactions on colliding cells trigger this repulsion, but how fibroblasts transition to clustering behaviour during healing remains unclear. Syndecan-4 modulates transmembrane receptors involved in wound healing, including receptors for the extracellular matrix and growth factors. As a result, *Sdc4*^–/–^ mice experience delayed healing due to impaired fibroblast recruitment. In this study, we report that syndecan-4 also regulates fibroblast repulsion during wound healing. We discover that syndecan-4 inhibits the expression and signalling of EphA2 by activating PKCα. Changes in syndecan-4 expression, such as those observed during wound healing, alter fibroblast behaviour from repulsion to adhesion upon cell collision by modulating EphA2 levels. Moreover, we find that EphA2 expression is suppressed in wound bed fibroblasts in a syndecan-4-dependent manner, explaining how fibroblast clustering is achieved during wound healing.

## Introduction

The UK spends £8.3 billion annually to treat 3.8 million individuals with healing defects, highlighting the significant burden that poor wound healing places on healthcare systems and patients' quality of life (Guest et al., 2020). Healing is achieved by responses at the cellular level that include an inflammatory response, wound contraction, and re-epithelialization. Failures in any of these steps lead to chronic wounds or pathological scarring. Fibroblasts play a critical role in wound contraction by clustering in the wound bed, secreting extracellular matrix (ECM) to form granulation tissue, and differentiating into myofibroblasts (Rognoni and Watt, 2018). Fibroblast migration towards a wound site is driven by polarized Rac1 activation, triggered by platelet-derived growth factor and fibronectin (Kwon et al., 2007; Liu et al., 2009; Barrientos et al., 2014). Syndecan-4 (SDC4), a co-receptor for growth factors and ECM, is crucial for guiding fibroblast migration via Rac1 regulation (Bass et al., 2007; Morgan et al., 2007). Consequently, *Sdc4*^–/–^ mice exhibit delayed healing, and fibroblasts from these mice have migration defects (Echtermeyer et al., 2001).

However, an important obstacle to fibroblast clustering has not been adequately addressed: contact inhibition of locomotion (CIL), which causes fibroblasts to repel each other, scattering throughout unwounded dermis (Batson et al., 2013). To cluster in the wound bed, fibroblasts must overcome this repulsive behaviour. CIL is initiated by interactions between Eph receptor tyrosine kinases (EphR) and membrane-tethered ephrins on adjacent cells, which trigger receptor clustering and bidirectional signalling that induces either membrane protrusion or retraction, depending on the EphRs and ephrins involved (Pasquale, 2008; Batson et al., 2013). In prostate cancer cells, EphB3 and EphB4 promote cell attraction, while EphA2 and EphA4 drive CIL via RhoA activation (Astin et al., 2010). Various EphRs are highly expressed in the skin, including EphA2, EphA4, EphB3, EphB4, and EphB6 (Hafner et al., 2006), with some contributing to wound healing. For example, expression of ephrinB1 and EphB2 rises in the wounded epidermis and loosens the junctions between keratinocytes to facilitate migration of the epithelium, and thus keratinocyte-specific knockout of ephrinB1/B2 leads to severely compromised skin healing (Nunan et al., 2015). Activation of EphB3/4 due to increased expression and shredding of the ligand, ephrinB2, stimulates skin fibroblast migration and differentiation into myofibroblasts during injury (Lagares et al., 2017). Furthermore, injection of soluble ephrinB2 ectodomain into a dermal wound induces fibrosis, while fibroblast-specific knockout of ephrinB2 inhibits differentiation into myofibroblasts and reduces collagen synthesis and fibrosis. High EphA expression has also been linked to non-healing wounds, with EphA1, EphA2, EphA4, and EphA7 all showing elevated levels in non-healing skin (Basu and Martins-Green, 2022), supporting the correlation between high EphA expression and poor healing.

While these studies highlight the importance of EphR expression and signalling during healing, the mechanisms controlling the switch from repulsive to adhesive fibroblast behaviour and how fibroblast residence in the wound bed is achieved remain unclear. In this study, we identify SDC4 as a key regulator of this switch. SDC4 is a receptor for molecules secreted upon injury, with its own expression controlled by the NF-κB pathway (Yang et al., 2015). Since SDC4 exhibits elevated expression during wound healing (Gallo et al., 1996), our results reveal an inverse relationship between SDC4 and EphA2 expression and signalling. High EphA2 levels in *Sdc4*^–/–^ MEFs result in heightened CIL, which is represented *in vivo* by the high EphA2 expression of poorly clustered fibroblasts in the wounded dermis of *Sdc4*^–/–^ mice. These findings suggest that SDC4 induction following injury switches fibroblasts from repulsive to adhesive behaviour, promoting wound contraction by allowing fibroblast clustering in the wound bed.

## Results

### Changes in SDC4 expression regulate expression of EphRs in an inverse manner

In unwounded skin, dermal fibroblasts repel one another through CIL, causing them to scatter. However, during wound healing, fibroblasts cluster to drive wound contraction. The mechanism behind this reversal in behaviour is unclear. Since SDC4 expression rises upon injury, given its critical role in coordinating various transmembrane receptors necessary for wound healing (Morgan et al., 2007), we investigated whether SDC4 influences CIL in fibroblasts.

**Figure 1:**
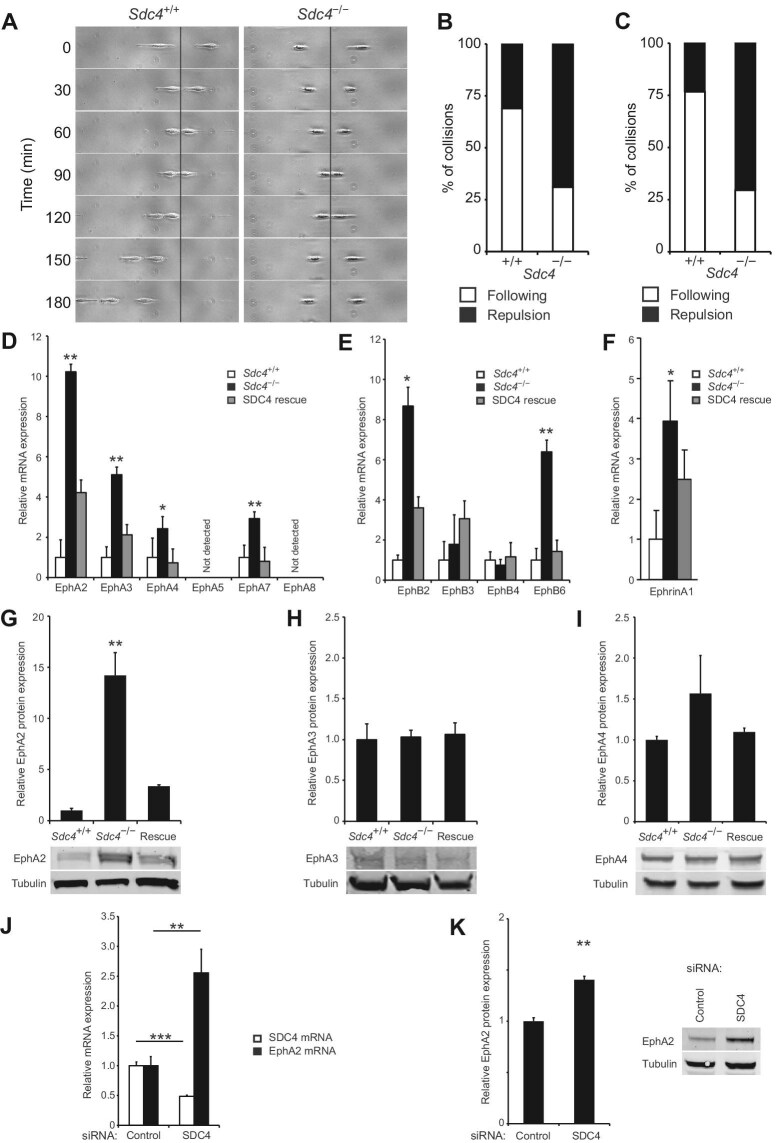
Expression of EphRs and cell repulsion are increased in the absence of SDC4. (**A**) Representative collisions between MEFs seeded on 5-μm fibronectin stripes (see Supplementary Videos S1 and S2). (**B**) Collisions were scored as ‘following’ if cells moved in the same direction after collision or ‘repulsion’ if cells moved in opposite directions (*n = *29). (**C**) Collisions, in the presence of 20 ng/ml TGFβ, were scored (*n = *34). (**D**–**F**) qPCR analysis of EphR and ephrin expression levels. Histograms depict fold changes relative to *Sdc4*^+/+^ MEFs from a representative experiment, with *n = *3 and experiments repeated up to 5 times. (**G**–**I**) Western blot analysis of EphA2 (**G**, *n = *3), EphA3 (**H**, *n = *3), and EphA4 (**I**, *n = *4) protein levels. (**J** and **K**) qPCR analysis of SDC4 and EphA2 mRNA levels (**J**, *n = *8) and western blot analysis of EphA2 protein level (**K**, *n* = 3) in MEFs transfected with non-targeting (Control) or SDC4-targeting (SDC4) siRNA. Error bars represent standard error; significance tested by analysis of variance (ANOVA); *P < 0.05, **P < 0.005, ***P < 0.0005.

Using a constrained migration assay, where fibroblasts were seeded onto 5-μm wide fibronectin stripes, narrow enough to prevent cells passing, collisions were classified as either ‘following’, with both cells moving in the same direction, or ‘repulsion’, with cells moving directly apart. The frequency of repulsion more than doubled in *Sdc4*^–/–^ MEFs, compared to *Sdc4*^+/+^ cells, demonstrating that SDC4 expression reduces cell–cell repulsion (Figure 1A and B; Supplementary Videos S1 and S2). Similar results were obtained in the presence of 20 ng/ml TGFβ, a key regulator of fibroblast function during healing (Figure 1C).

EphR signals are key mediators of transient cell–cell interactions and were examined to determine whether SDC4 influences their expression. Through quantitative polymerase chain reaction (qPCR) analysis, we found that messenger RNA (mRNA) levels of various EphRs were elevated in *Sdc4*^–/–^ MEFs compared to *Sdc4*^+/+^ cells, and these levels were suppressed upon re-expression of SDC4 (Figure 1D and E). Among these, EphA2 was most notably affected and its preferred ligand, ephrinA1 (Koolpe et al., 2002), also showed significantly elevated expression in *Sdc4*^–/–^ MEFs (Figure 1F). EphB2 expression also increased, which is notable as it affects epidermal migration during wound healing (Nunan et al., 2015). Protein level analyses confirmed elevated EphA2 in *Sdc4*^–/–^ MEFs (Figure 1G). EphA3 expression was low in the skin (Hafner et al., 2006; Figure 1H) and EphA4 was less impacted by SDC4 at the protein level (Figure 1I). EphA2 mRNA and protein levels also increased upon transient knockdown of SDC4 using small-interfering RNA (siRNA) (Figure 1J and K), reinforcing the regulatory role of SDC4 over EphA2.

**Figure 2:**
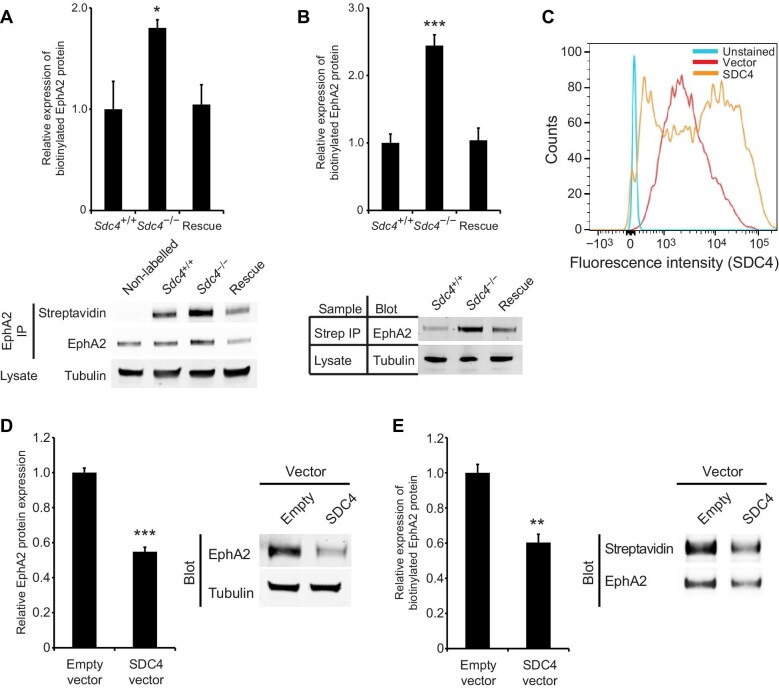
Reciprocal relationship between expression of SDC4 and surface expression of EphA2. (**A** and **B**) MEFs were surface-labelled with biotin before immunoprecipitating EphA2 and probing with fluorophore-conjugated streptavidin (**A**, *n = *3) or precipitating biotinylated proteins and blotting for EphA2 (**B**, *n = *7). (**C**–**E**) SDC4 was overexpressed in MEFs via retroviral infection with the SDC4 cDNA, using empty vector as a negative control. (**C**) SDC4 expression in infected MEFs by flow cytometry. (**D**) Western blot analysis of EphA2 protein levels in cell lysates (*n = *10). (**E**) Surface EphA2 protein levels by immunoprecipitating EphA2 from MEFs surface-labelled with biotin and probing with fluorophore-conjugated streptavidin (*n = *5). Error bars represent standard error; significance tested by ANOVA; *P < 0.05, **P < 0.005, ***P < 0.0005.

Alterations in EphA2 mRNA and protein levels resulted in a corresponding change in surface protein levels. MEFs were biotinylated in culture, before washing away free biotin and detecting the labelled cell-surface protein by either immunoprecipitating EphA2 and probing with streptavidin (Figure 2A) or precipitating biotin-labelled protein and probing the isolate for EphA2 (Figure 2B). Regardless of approach, surface levels of EphA2 were found to be elevated in *Sdc4*^–/–^ MEFs compared to *Sdc4*^+/+^ cells and suppressed by stable re-expression of SDC4.

During healing, SDC4 expression rises (Gallo et al., 1996), and our proposal is that the elevation of SDC4 encourages fibroblast clustering by suppressing EphA2. To test whether rises in SDC4 expression do inhibit EphA2 expression, *Sdc4*^+/+^ MEFs were infected with retroviral virions containing the SDC4 cDNA or empty vector. SDC4 overexpression, verified by flow cytometry (Figure 2C), led to reduced total and surface EphA2 expression (Figure 2D and E), demonstrating that SDC4 not only influences EphA2 at normal expression levels but also suppresses EphA2 expression when SDC4 levels are elevated, as that occurs during wound healing. Collectively, these results demonstrate that SDC4 expression regulates EphA2 at the mRNA, total protein, and surface expression levels.

### SDC4 expression influences EphA2 signalling

**Figure 3:**
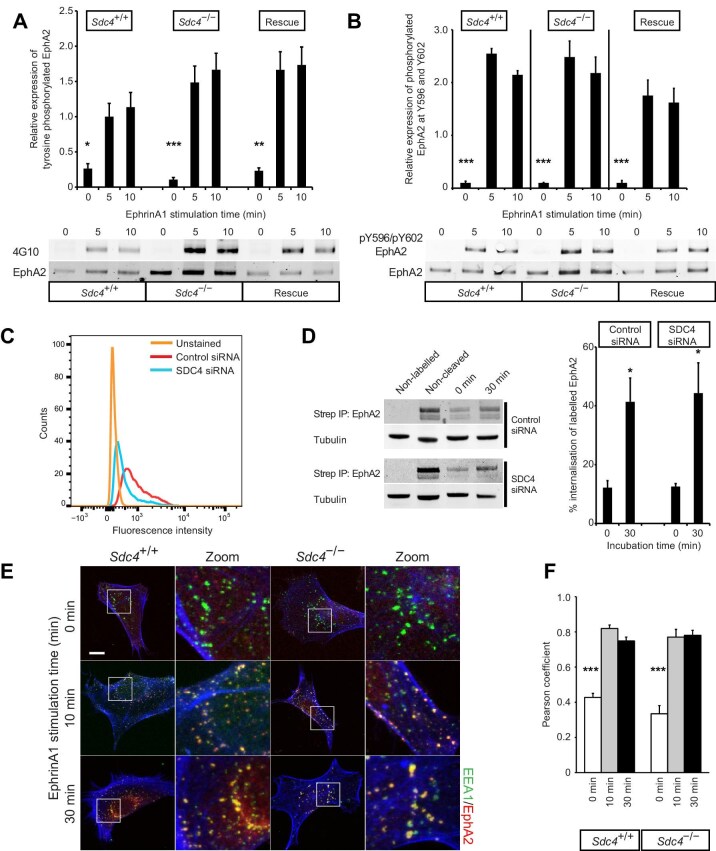
Phosphorylation and endocytosis of EphA2 increase proportionally to expression. (**A** and **B**) MEFs were stimulated with 1 μg/ml clustered ephrinA1-Fc, followed by western blot analysis using antibody 4G10 for total phosphotyrosine (**A**, *n = *4) or an antibody against EphA2 residues Y596 and Y602 (**B**, *n = *4). Histograms represent phosphorylation levels normalised to EphA2. (**C** and **D**) EphA2 endocytosis in fibroblasts transfected with non-targeting (Control) or SDC4-targeting (SDC4) siRNA. (**C**) Flow cytometry analysis of SDC4 expression. (**D**) Fibroblasts were surface-labelled with biotin and stimulated with 1 μg/ml clustered ephrinA1-Fc. The internalised proteins were precipitated with streptavidin before blotting for EphA2 (*n = *6). (**E** and **F**) MEFs were stimulated with 1 μg/ml clustered ephrinA1-Fc before fixation and staining (see also Supplementary Figure S1). (**E**) Representative images of MEFs stained for EEA1 (green), EphA2 (red), and counterstained with phalloidin (blue). Scale bar, 10 μm. (**F**) Pearson correlation analysis for colocalization between EphA2 and EEA1 (*n = *10). Error bars represent standard error; significance tested by ANOVA; *P < 0.05, **P < 0.005, ***P < 0.0005.

Upon ephrin binding, EphRs cluster, autophosphorylate, and undergo endocytosis, signalling from both the plasma membrane and endocytic vesicles. The increased reciprocal repulsion observed in *Sdc4*^–/–^ MEFs suggests an elevation in Eph signalling, phosphorylation, and endocytosis, corresponding to the elevated expression. To investigate this, MEFs were stimulated with clustered, Fc-tagged ephrinA1 (EphA2’s preferred ligand), and immunoprecipitated EphA2 was blotted for phosphotyrosine using the 4G10 antibody. In *Sdc4*^–/–^ MEFs, the amount of tyrosine-phosphorylated EphA2 increased compared to *Sdc4*^+/+^ or rescued cells, correlating with higher EphA2 expression and demonstrating that the highly expressed EphA2 could be activated (Figure 3A). Phosphoproteomic studies have shown that phosphorylation of the juxtamembrane region of EphRs precedes and is necessary for activation of the EphR kinase domain (Singla et al., 2011). Probing immunoprecipitated EphA2 for the phosphorylated forms of the conserved Y596 and Y602 juxtamembrane tyrosines revealed that phosphorylation of these residues was also proportional to EphA2 abundance (Figure 3B), indicating that the elevated EphA2 in *Sdc4*^–/–^ MEFs result in a corresponding increase in active receptor upon ligand engagement.

EphA2 phosphorylation is followed by endocytosis (Greene et al., 2014), which is crucial for cell repulsion as it converts initial adhesion into repulsion (Valenzuela and Perez, 2020). To assess whether the highly expressed EphA2 is proportionately endocytosed and exhibits elevated signalling, fibroblasts were surface-biotinylated, stimulated with clustered ephrinA1-Fc, and then residual surface biotin was cleaved. Internalized receptors were affinity-precipitated with streptavidin and blotted for EphA2. Endocytosis was tested in human fibroblasts transfected with control or EphA2-targeting siRNA because MEFs could not withstand the biotinylation and cleavage steps. Similar proportions of EphA2 were endocytosed in both control and SDC4-knockdown fibroblasts, demonstrating that endocytosis is proportional to EphA2 expression (Figure 3C and D). Vesicular uptake of EphA2 was also demonstrated by immunofluorescence. Upon ephrinA1-Fc stimulation, EphA2 translocated into EEA1-positive vesicles in both *Sdc4*^–/–^ and *Sdc4*^+/+^ MEFs, with similar Pearson correlations between the groups in *Sdc4*^–/–^, *Sdc4*^+/+^, and rescued MEFs (Figure 3E and F; Supplementary Figure S1). EphA2’s progression into Rab11-positive recycling endosomes was also comparable between *Sdc4*^+/+^ and *Sdc4*^–/–^ MEFs (Supplementary Figure S2), demonstrating that the highly expressed EphA2 in *Sdc4*^–/–^ MEFs is fully functional and likely increases their sensitivity to ephrinA1.

**Figure 4:**
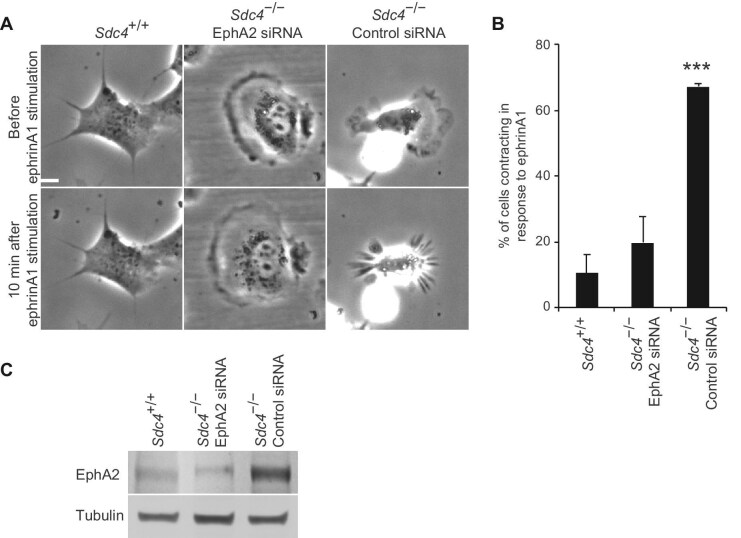
*Sdc4* ^–/–^ MEFs contract in response to ephrinA1. *Sdc4*^+/+^ and *Sdc4*^–/–^ MEFs transfected with either non-targeting (Control) or EphA2-targeting (EphA2) siRNA were spread on 5 μg/ml fibronectin-coated dishes and stimulated with 1 μg/ml clustered ephrinA1-Fc. (**A**) Representative frames before and after the addition of ephrinA1-Fc (see Supplementary Videos S3–S5). Scale bar, 20 μm. (**B**) Percentage of cells that contracted upon the addition of ephrinA1-Fc. The histogram represents the average of 3 experiments, with a total of 47 cells of each type scored. (**C**) Western blot analysis of EphA2 expression in cells used for the contraction assay and subsequent migration assay. Error bars indicate standard error; significance tested by ANOVA; ***P < 0.0005.

To test the hypothesis that *Sdc4*^–/–^ MEFs are more sensitive to ephrin, cell contraction was monitored in fibroblasts spread on fibronectin. All cell types remained fully spread prior to ephrinA1-Fc stimulation (Supplementary Videos S3–S5). Following stimulation, most *Sdc4*^–/–^ MEFs contracted, whereas only a few *Sdc4*^+/+^ MEFs did so (Figure 4A and B; Supplementary Videos S3–S5), confirming the heightened sensitivity of *Sdc4*^–/–^ MEFs to ephrinA1, consistent with the elevated levels of active EphA2 (Figure 3A and B).

A critical question was whether the behaviour of *Sdc4*^–/–^ MEFs is solely due to elevated EphA2 expression or attributed to other EphRs (Figure 1) or reduced adhesive properties, since SDC4 promotes focal adhesion formation (Bass et al., 2007). To address this, siRNA was used to knock down EphA2 levels in *Sdc4*^–/–^ MEFs comparable to that in *Sdc4*^+/+^ MEFs (Figure 4C). This blocked ephrinA1-stimulated contraction (Figure 4A and B; Supplementary Video S4), demonstrating that the heightened sensitivity of *Sdc4*^–/–^ MEFs is indeed mediated by EphA2 and confirming SDC4’s role in modulating EphA2 signalling during cell repulsion.

### SDC4 regulates EphA2 expression by PKCα activation

**Figure 5:**
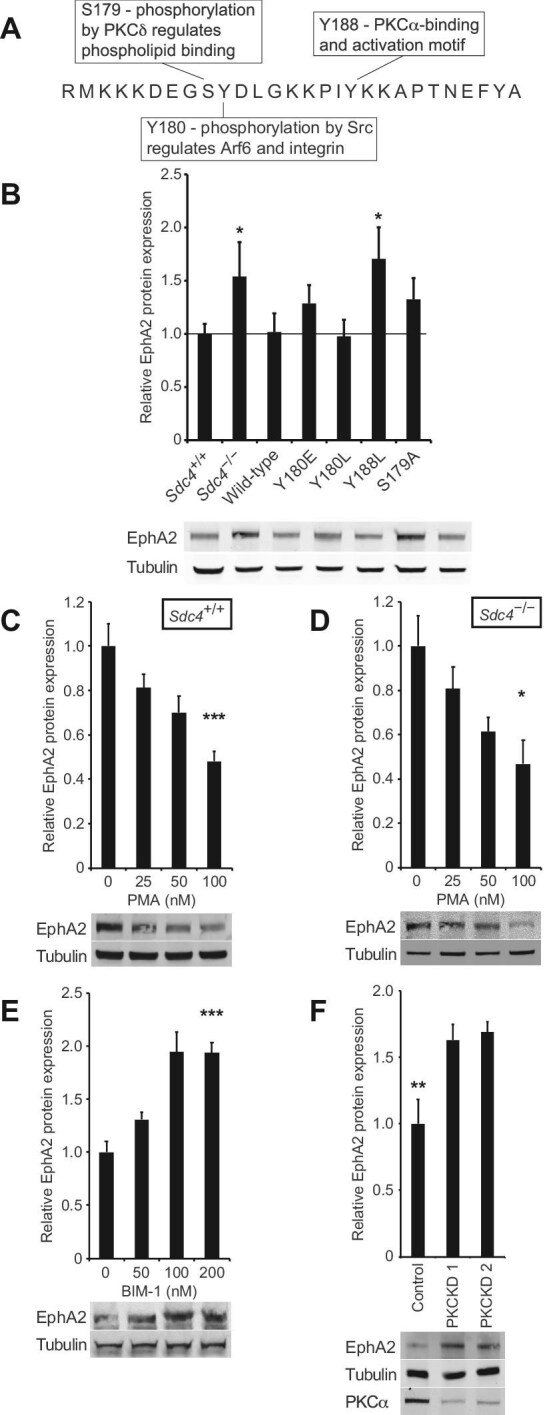
SDC4 regulates EphA2 expression by activation of PKCα. (**A**) SDC4 cytodomain indicating the functions of residues to be tested. (**B**–**F**) Western blot analysis of EphA2 protein levels in *Sdc4*^+/+^ MEFs, *Sdc4*^–/–^ MEFs, and *Sdc4*^–/–^ MEFs stably rescued with the indicated SDC4 cDNAs (**B**, *n = *5), *Sdc4*^+/+^ (**C**, *n = *10) and *Sdc4*^–/–^ (**D**, *n = *4) MEFs treated daily with indicated concentrations of PMA for 5 days, *Sdc4*^+/+^ MEFs treated daily with indicated concentrations of the PKCα inhibitor, BIM-1, for 5 days (**E**, *n = *6), and *Sdc4*^+/+^ MEFs transfected with control or PKCα-targeting siRNA (**F**, *n = *6). Error bars represent standard error; significance tested by ANOVA; *P < 0.05, **P < 0.005, ***P < 0.0005.

The cytoplasmic domain of SDC4 is known to mediate various cellular processes, such as Rac1-dependent migration guidance (Bass et al., 2007), receptor endocytosis (Bass et al., 2011), and receptor recycling (Morgan et al., 2013), through specific functional motifs. To understand which part of the SDC4 cytoplasmic domain regulates EphA2 expression, *Sdc4*^–/–^ lines were engineered to stably re-express different SDC4 mutants with known functional defects (Figure 5A). Wild-type SDC4 re-expression restored EphA2 expression levels in *Sdc4*^–/–^ MEFs comparable with that in *Sdc4*^+/+^ cells (Figure 5B), confirming SDC4’s role in regulating EphA2.

The key interaction of SDC4 involves binding to and activating PKCα, an effect that can be disrupted by a Y188L mutation in the SDC4 cytoplasmic domain (Koo et al., 2006). Re-expression of the SDC4-Y188L mutant failed to restore EphA2 levels in *Sdc4*^–/–^ MEFs, demonstrating that the PKCα-binding motif is essential for EphA2 regulation and PKCα activation suppresses EphA2 expression (Figure 5B). Like *Sdc4*^+/+^ and *Sdc4*^–/–^ MEFs (Figure 3A), MEFs expressing the Y188L mutant still activated EphA2 in response to ephrinA1 (Supplementary Figure S3A). Mutants S179A and Y180E, previously reported to regulate oligomerization of the SDC4 cytoplasmic domains and thus attenuate PKCα binding without blocking it entirely (Koo et al., 2006), only partially restored EphA2 levels (Figure 5B). These results support that the level of PKCα binding is critical for proper suppression of EphA2 by SDC4.

To test whether PKCα mediates the regulation of EphA2 by SDC4, PKCα activity was manipulated by using phorbol 12-myristate 13-acetate (PMA) to activate PKCα or bisindolylmaleimide-1 (BIM-1) to inhibit it. Daily treatment with PMA for 5 days, at increasing concentrations, suppressed EphA2 expression in both *Sdc4*^+/+^ and *Sdc4*^–/–^ MEFs, demonstrating that PKCα activation downregulates EphA2 (Figure 5C and D). Conversely, inhibition of PKCα for 5 days using BIM-1 increased EphA2 expression in *Sdc4*^+/+^ MEFs (Figure 5E). As a control for the treatment regime, PKC activity was compared between conditions by blotting whole lysates with an antibody against the consensus phospho-serine PKC substrate motif. PMA-treated cells showed an increase in phosphorylation of a number of bands, while BIM-1 reduced it, confirming the modulation of PKC activity (Supplementary Figure S3B). Additionally, basal phosphorylation levels were lower in *Sdc4*^–/–^ than in *Sdc4*^+/+^ MEFs, consistent with the reduced PKCα signalling in *Sdc4*^–/–^ MEFs. Finally, knockdown of PKCα expression using two different siRNAs resulted in elevated EphA2 expression (Figure 5F), ruling out off-target effects of the pharmacological inhibitors. Collectively, these findings demonstrate that SDC4 regulates EphA2 expression by activating PKCα and the inhibition of this pathway at any step leads to an increase in EphA2 levels.

### SDC4 and EphA2 form a functional relationship in skin healing

**Figure 6:**
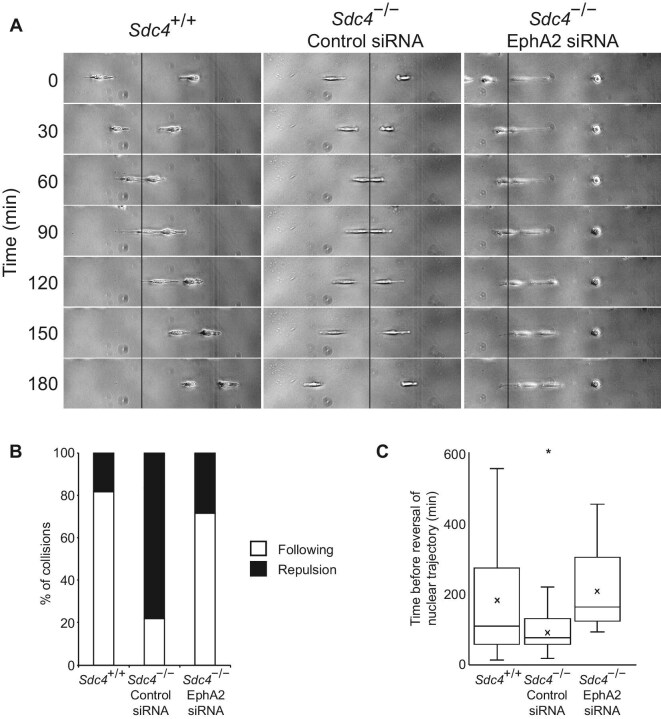
Homotypic repulsion of *Sdc4*^–/–^ MEFs is due to high EphA2 expression. (**A**) Representative collisions between *Sdc4*^+/+^ (*n = *93) and *Sdc4*^–/–^ MEFs transfected with either non-targeting (Control, *n = *78) or EphA2-targeting (EphA2, *n = *14) siRNA (see Supplementary Videos S6–S8). (**B**) Collisions were scored as ‘following’ or ‘repulsion’. (**C**) Time before both nuclei made a retrograde step post-collision. Boxes indicate median and 1st and 3rd quartiles and whiskers indicate data range. Significance tested by ANOVA; *P < 0.05.

The *in vitro* experiments established that SDC4 regulates EphA2 expression and activation, which is hypothesized to explain the mutual repulsion observed in *Sdc4*^–/–^ MEFs. To directly test the hypothesis, siRNA was used to reduce EphA2 expression levels in *Sdc4*^–/–^ MEFs to match that in *Sdc4*^+/+^ MEFs (Figure 4C). In a constrained migration assay, the frequency of repulsion more than doubled in *Sdc4*^–/–^ MEFs transfected with non-targeting siRNA, compared to *Sdc4*^+/+^ MEFs, while the repulsion frequency in *Sdc4*^–/–^ MEFs transfected with siRNA targeting EphA2 was similar to that in *Sdc4*^+/+^ cells (Figure 6A and B), indicating that SDC4-dependent EphA2 expression plays a critical role in mediating cell repulsion.

To further investigate the impact of cell–cell collision on migration behaviour, the time for the nuclei of both colliding cells to take retrograde steps from the contact point was recorded. *Sdc4*^–/–^ MEFs responded more rapidly, while *Sdc4*^+/+^ cells and *Sdc4*^–/–^ cells with reduced EphA2 (via siRNA) showed a slower reversal of nuclei (Figure 6C). This indicates that EphA2 expression, controlled by SDC4, influences the degree of fibroblast repulsion.

**Figure 7:**
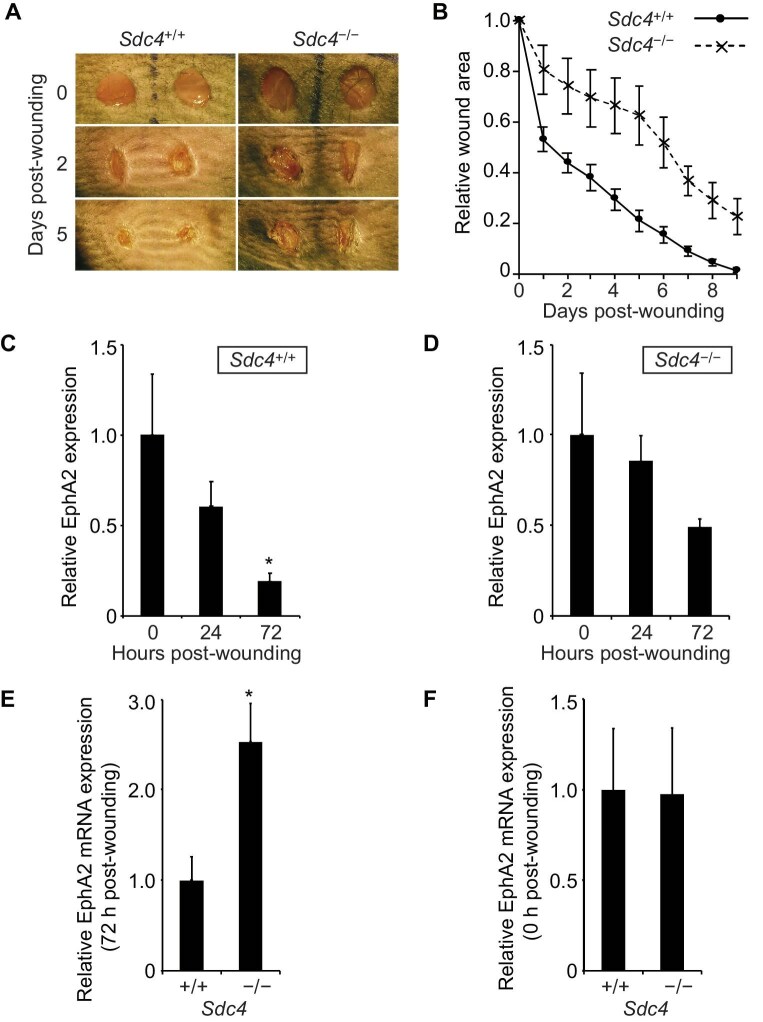
EphA2 expression in the fibroblasts of wounded skin is regulated by SDC4. (**A** and **B**) In mouse wound healing model, 4-mm full-thickness wounds were created on the backs of mice, and wound areas were recorded macroscopically. (**C**–**E**) qPCR analysis of EphA2 mRNA expression levels, relative to 18S internal control, in the bed of full-thickness skin wounds. Shown are relative expression changes over time in *Sdc4*^+/+^ (**C**) and *Sdc4*^–/–^ (**D**) mice and direct comparison between two genotypes at 72 h (**E**) or 0 h (**F**) post-wounding (*n = *9). Error bars represent standard error; significance tested by ANOVA; *P < 0.05.

A knockout mouse model was employed to study whether SDC4 regulates EphA2 expression during wound healing *in vivo. Sdc4*^–/–^ mice, which received 4-mm punch biopsies, exhibited slower wound healing compared to *Sdc4*^+/+^ mice (Figure 7A and B). When wound bed samples were analysed via qPCR, EphA2 expression in *Sdc4*^+/+^ mice was found to decrease significantly within 72 h post-wounding, paralleling the rise in SDC4 levels (Gallo et al., 1996). This reduction in EphA2 benefitted fibroblast clustering by reducing cell repulsion (Figure 7C). In contrast, any EphA2 suppression in *Sdc4*^–/–^ mice was weaker and not significant (Figure 7D). Although we cannot definitively rule out weaker EphA2 suppression by other mechanisms, *Sdc4*^–/–^ mice exhibited significantly higher EphA2 expression at 72 h post-wounding compared to *Sdc4*^+/+^ mice (Figure 7E). In unwounded skin, where SDC4 expression is typically low, EphA2 expression levels were similar in *Sdc4*^+/+^ and *Sdc4*^–/–^ mice (Figure 7F).

In summary, the relationship between SDC4 and EphA2 explains how fibroblasts suppress scattering behaviour in response to wounding, facilitating more efficient cell clustering, and wound repair. This functional interaction highlights the importance of SDC4 in modulating EphA2-mediated repulsion and its critical role in skin healing processes.

## Discussion

Here, we report the suppression of EphA2 expression by SDC4, a process that directly affects fibroblast interactions upon contact. Previous studies have shown that EphA2 regulates fibronectin deposition and ECM adhesion maturation (Finney et al., 2021). Our findings now reveal that SDC4, a fibronectin receptor, controls EphA2 expression, establishing a bidirectional relationship between ECM receptors and cell–cell receptors. This interaction explains the modulation of fibroblast behaviour during wound healing.

We demonstrate that SDC4 regulates EphA2 expression via its interaction with PKCα. Substitution of the PKCα-binding site in SDC4 or inhibition of PKCα resulted in elevated EphA2 expression, while activation of PKCα using PMA suppressed EphA2 expression (Figure 5). While PKC's role in gene regulation is well known, acting through pathways such as PMA-responsive elements in gene promoters, ERK, NF-κB, and JAK/STAT pathways (Caino et al., 2011), the regulation of transcription via PKCα might not be direct. SDC4 regulates cytoskeletal rearrangement and stress fibre formation in a PKCα-dependent manner (Morgan et al., 2007), and tension in the actin cytoskeleton is known to regulate transcription through YAP/TAZ (Dupont et al., 2011). PKCα has been shown to exert both positive and negative influence on expression of 19 different Gene Ontology gene sets that include several transmembrane protein groups (Caino et al., 2011). The range of PKCα targets, encompassing both direct kinase substrates and downstream effects on gene expression, has historically made it difficult to clearly define the physiological role of PKCα and understand how specificity is achieved. Activation of PKCα by SDC4 is site-specific, as the two proteins interact at particular cellular locations, thus conferring specificity to PKCα signalling. The broad specificity of PKCα as a kinase, combined with the range of genes it regulates, could suggest that the SDC4/PKCα interaction might influence other receptor systems as well.

EphA2’s role in CIL is mediated by phosphorylation and endocytosis upon cell collision (Greene et al., 2014; Valenzuela and Perez, 2020). Receptor endocytosis converts the adhesive nature of an EphR/ephrin interaction into a repulsive one, making it a crucial event (Marston et al., 2003; Boissier et al., 2013). Our data show that SDC4’s influence on EphA2 expression is reflected in the phosphorylation and endocytosis of the receptor, establishing a link between SDC4 and CIL in fibroblasts. EphA2 endocytosis both triggers and depends on the activation of Rac1 (Zhuang et al., 2007; Boissier et al., 2013). SDC4 also regulates Rac1-dependent migration guidance (Bass et al., 2007; Matthews et al., 2008), though the mechanism by which this is achieved has not been resolved. Rac1 activity is constitutively high in *Sdc4*^–/–^ MEFs and has been proven to be responsible for the migratory behaviour of individual *Sdc4*^–/–^ MEFs (Bass et al., 2007). The elevated EphA2 expression and activity in *Sdc4*^–/–^ MEFs may contribute to the dysregulated Rac1 activity observed in these cells, affecting their migratory behaviour.

Following EphA2 endocytosis, RhoA activation occurs through the binding of exchange factors like Vav2 and ephexin1 to activated EphA2/ephrinA1 on endosomes (Shamah et al., 2001; Batson et al., 2014). RhoA activation causes membrane retraction and cell repulsion and is responsible for the switch from protrusion to CIL (Batson et al., 2014). SDC4 also influences RhoA by first inhibiting it through p190RhoGAP and later activating it, a pattern resembling ECM-induced spreading (Bass et al., 2008; Dovas et al., 2010). EphA2 and SDC4 signals converge at p190RhoGAP, with EphA2 modulating its tyrosine phosphorylation (Finney et al., 2021) and SDC4 stimulating serine/threonine phosphorylation (Bass et al., 2008), with the result that the two receptors jointly regulate RhoA-mediated contractility. SDC4 also regulates integrin-mediated adhesion, enhancing adhesion formation and modulating integrin endocytosis (Morgan et al., 2007; Bass et al., 2011), highlighting the integrative role of SDC4 in coordinating cell–ECM and cell–cell interactions.

Key signals including Rac1 and RhoA depend on the balance between cell–ECM and cell–cell interactions. The suppression of EphA2 by SDC4 as we report, combined with the previously reported effect of SDC4 on integrins (Bass et al., 2011), means that SDC4 plays a central role in balancing cell–ECM and cell–cell signals and creating a coherent response to changes in the physiological environment. This balance is crucial for fibroblast clustering and migration during wound healing and could also extend to other cell types and tissues. EphA2 is highly expressed in a wide range of tissues, including the colon, kidney, and bladder, and endothelial cells (Hafner et al., 2006; Vreeken et al., 2020). While the specific profiles of EphR/ephrin expression vary by tissue, the principles of SDC4 regulation may still apply. For instance, cadherin-mediated cell–cell junctions play a crucial role in epithelial and endothelial cells by opposing cell separation and repulsion. VE-cadherin endocytosis is impaired in the endothelial cells of *Sdc4*^–/–^ mice resulting in defective angiogenesis (De Rossi et al., 2021). Although the underlying mechanisms vary significantly between different tissue types, these observations underscore the essential role of SDC4 in regulating cell–cell interactions, particularly during healing responses.

The importance of cell regulators such as SDC4 is underscored by the role of such molecules in preventing excessive cellular infiltration, a hallmark of invasive cancer. Our discovery that SDC4 controls EphA2 at the expression level, rather than merely at the activity level, opens up new research avenues. If SDC4 similarly regulates the expression of other receptors, such as integrins and growth factor receptors, it could serve as a master regulator of cell behaviour, integrating the surface expression of receptors with complementary or antagonistic functions and coordinating cell responses to their microenvironment.

This study provides significant insight into the regulatory mechanisms that govern cell–cell interactions, migration, and wound healing, highlighting SDC4 as a critical mediator of these processes. Further research into the broader implications of SDC4’s regulation of receptor expression could have profound implications for understanding tissue homeostasis, wound repair, and pathological conditions such as cancer.

## Materials and methods

### qPCR

RNA was extracted from cells or skin biopsies using TRI Reagent (Supplementary Materials and methods). Then, 5 mg RNA was treated with DNase (Roche) before generating cDNA using a Maxima First Strand cDNA Synthesis Kit for qRT-PCR (Fermentas). qPCR was conducted using the DNA Engine Opticon^®^2 System (Bio-Rad), using Maxima SYBR Green Master Mix (Fermentas) and Quantitect Primers (Qiagen). The Comparative Ct (ΔΔCt) method was used to calculate changes in gene expression, normalizing Ct values to an 18S standard before calculating fold change over wild-type.

### Surface labelling and immunoprecipitation

Cells were surface-labelled with 0.13 mg/ml sulpho-NHS-LC-biotin (ThermoFisher) for 30 min at 4°C, before quenching with 1 M Tris (pH 6.8) and lysing in 50 mM Tris (pH 7.5), 250 mM sodium chloride, 10 mM magnesium chloride, 5 mM EGTA, 1% Triton X-100, 0.5% sodium deoxycholate, and Complete Protease Inhibitor (Roche). Protein was either immunoprecipitated with anti-EphA2 antibody (R&D Systems, AF639) and Dynabeads™ Protein G (ThermoFisher) before western blotting with DyLight800-conjugated streptavidin (ThermoFisher), for phosphotyrosine (4g10, Millipore) or pY596/pY602 EphA2 (Marston et al., 2003), or precipitated with Dynabeads™ Streptavidin (ThermoFisher) before western blotting for EphA2.

### Clustered ephrinA1

EphrinA1-Fc (R&D Systems) was pre-clustered before use with anti-human IgG (Stratech Scientific) for 10 min at 37°C.

### Internalization

Cells were starved for 2 h at 37°C in serum-free medium before surface labelling with 0.2 mg/ml sulpho-NHS-SS-biotin (ThermoFisher) in cold phosphate-buffered saline (PBS) for 30 min at 4°C, followed by washing with 100 mM glycine in PBS. Pre-warmed, serum-free medium containing 0.6 mM primaquine (Sigma) to inhibit recycling and 1 μg/ml clustered ephrinA1 was added to the cells, which were then incubated at 37°C for the required receptor internalization time. After incubation, cells were transferred to ice and washed twice with ice-cold PBS before removing biotin from proteins remaining on the cell surface with 20 mM sodium 2-mercaptoethanesulfonate (MesNa) (Sigma), 50 mM Tris (pH 8.6), and 100 mM NaCl for 15 min at 4°C. The cells were washed twice with ice-cold PBS, and residual MesNa was quenched with 20 mM iodoacetamide (Sigma), 50 mM Tris (pH 8.6), and 100 mM NaCl for 10 min at 4°C. After another PBS wash, cells were lysed in 50 mM Tris (pH 7.5), 250 mM NaCl, 10 mM MgCl_2_, 5 mM EGTA, 1% Triton X-100, 0.5% deoxycholate, 0.1% sodium dodecyl sulphate, and Complete Protease Inhibitor (Roche). Biotinylated proteins were precipitated with Dynabeads™ Streptavidin (ThermoFisher) and analysed by western blotting for EphA2 (R&D Systems AF639).

### Immufluorescence

Cells cultured on glass coverslips were stimulated with 1 μg/ml clustered ephrinA1, followed by fixation with 4% formaldehyde, quenching with 0.1 M glycine, and permeabilization with 0.4% Triton X-100 in PBS where appropriate. Cells were blocked with 3% bovine serum albumin in PBS and stained for EphA2 (R&D Systems) and EEA1 (BD Transduction Labs) using Alexa488- and Alexa594-conjugated IgGs (Stratech Scientific), along with Atto647N-conjugated phalloidin (Sigma). Images were captured using a Leica TCS SP5 II confocal microscope with a 100× objective (N/A 1.4).

### Cell contraction

MEFs were spread on 5 μg/ml fibronectin (Sigma) for 2 h at 37°C before being stimulated with 3 μg/ml clustered ephrinA1. Time-lapse images were captured at 30-sec intervals for 10 min before and after stimulation using an inverted Zeiss microscope at 37°C with 5% CO_2_, equipped with an Orca-ER camera (Hamamatsu) and Volocity software.

### Migration

MEFs were spread in Fibroblast Basal Media (ThermoFisher) in individual wells on a 20 × 20 mm² CYTOOchip (CYTOO #30-011) for 2 h at 37°C before capturing time-lapse images at 5-min intervals using an inverted Leica DMIRE2 microscope at 37°C with 5% CO_2_ for up to 16 h. Movie analysis was performed using ImageJ, and cell–cell collisions were classified as either ‘following’ or ‘repulsion’ by visual assessment. The time between cell–cell contact and the nuclei of both cells moving away from the contact point in consecutive frames was recorded. For each collision, cells were required to have been free of contact with other cells for 50 min prior.

### Mouse wound healing

Animal experiments were conducted in accordance with Home Office regulations under the UK Animals (Scientific Procedures) Act 1986, project license 30/2791. *Sdc4*^+/+^ and *Sdc4*^–/–^ C57BL/6J mice (Ishiguro et al., 2000) were generated from heterozygous (*Sdc4*^+/–^C57BL/6J) parental crosses. Twelve-week-old mice were anesthetised with isoflurane inhalation, and full-thickness excisional wounds were made on the shaved back, on either side of the dorsal midline, using a 4-mm biopsy punch (Kai Industries). Wound closure was recorded macroscopically by photographing the live mice daily until the wounds had closed, with images analysed using ImageJ. For qPCR analysis, biopsies were collected at 0, 24, and 72 h post-wounding.

## Supplementary Material

mjae054_Supplemental_Files
