# Supplementary material for: Inhibition of EphA2 by syndecan-4 in wounded skin regulates clustering of fibroblasts
Source: J Mol Cell Biol. 2024 Dec 23;17(1):mjae054. doi: 10.1093/jmcb/mjae054 (PMC12205308; doi:10.1093/jmcb/mjae054)
Supplement: mjae054_Supplemental_Files [file mjae054_supplemental_files.zip › Supplementary Material.pdf]

# Supplementary data

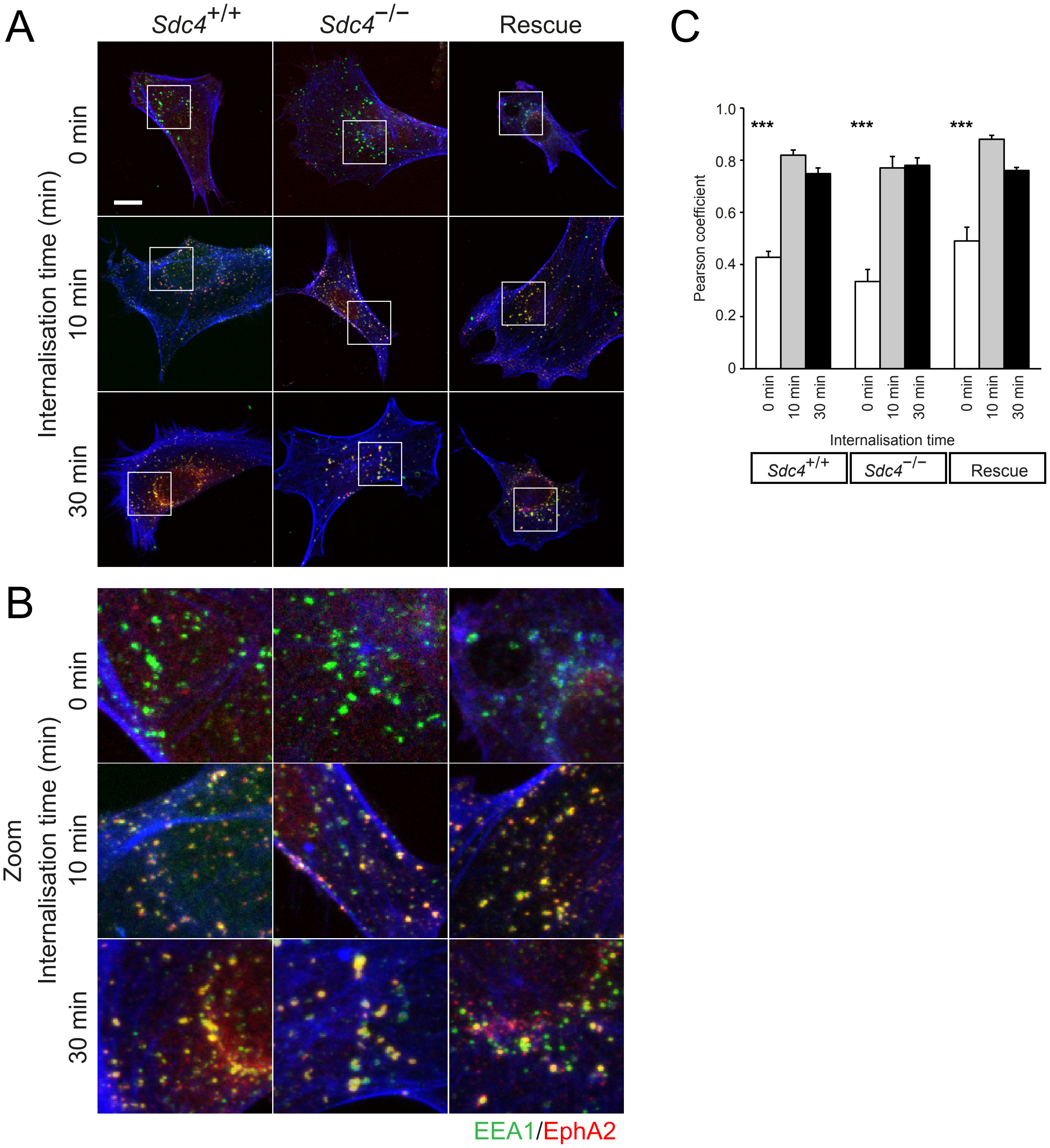


**Supplementary Figure S1. Colocalisation of endocytosed EphA2 and EEA1.**

*Sdc4*+/+, *Sdc4*–/–, and *Sdc4*–/– stably rescued with SDC4 MEFs were stimulated with 1 µg/ml clustered ephrinA1-Fc before fixing and staining. (A+B) Representative images of MEFs stained for EEA1 (green), EphA2 (red), and counterstained with phalloidin (blue). White boxes in (A) delineate the cropped area depicted in (B). (C) Pearson correlation for colocalisation between EphA2 and EEA1, n=10. Bar = 10 µm, error bars represent standard error, significance was tested by ANOVA, * $p<0.05$, ** $p<0.005$, *** $p<0.0005$. Partially reproduced in Figure 3.

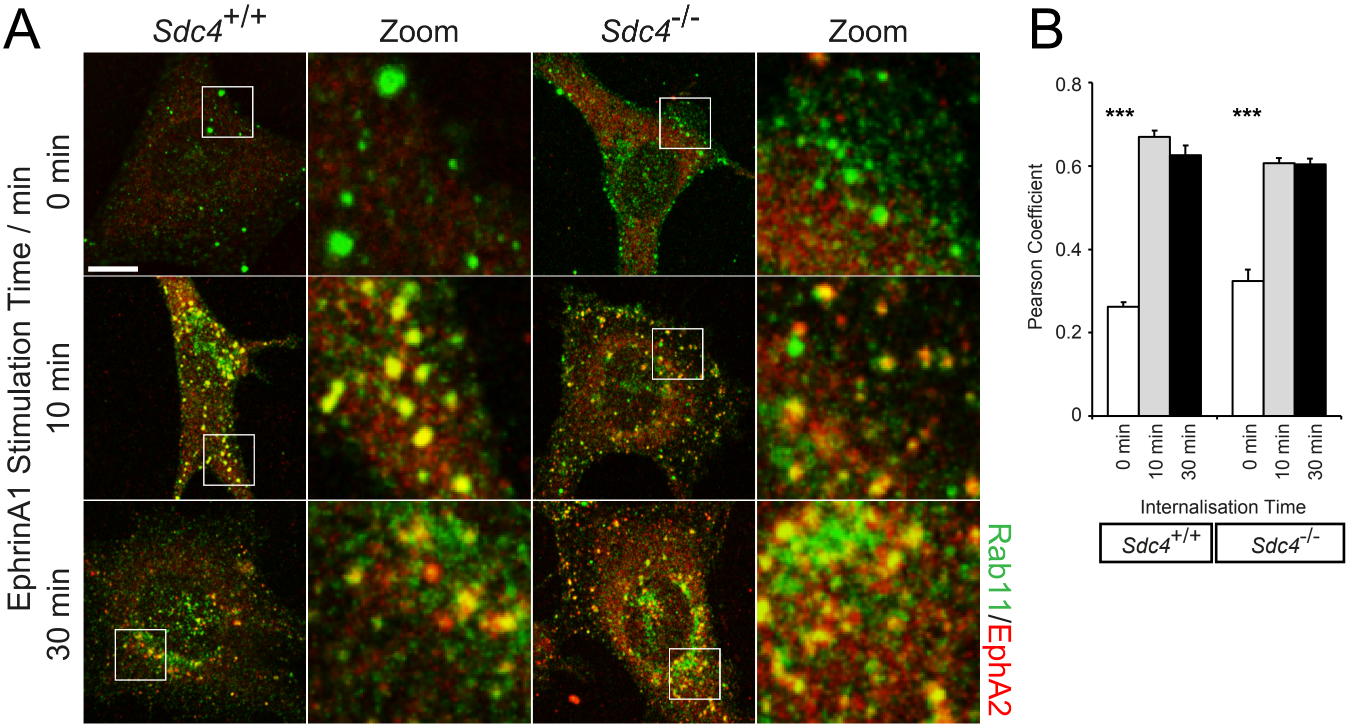


**Supplementary Figure S2. Stimulated EphA2 passes through similar trafficking pathways in Sdc4+/+ and Sdc4–/– MEFs.**

(A+B) *Sdc4*$^{+/+}$ and *Sdc4*$^{–/–}$ MEFs were stimulated with 1 µg/ml clustered ephrinA1-Fc before fixing and staining. (A) Representative images of MEFs stained for Rab11 (green) and EphA2 (red). Bar = 10 µm. (B) Pearson correlation for colocalisation between EphA2 and Rab11, n=30. Error bars represent standard error, significance was tested by ANOVA, *** $p<0.0005$.

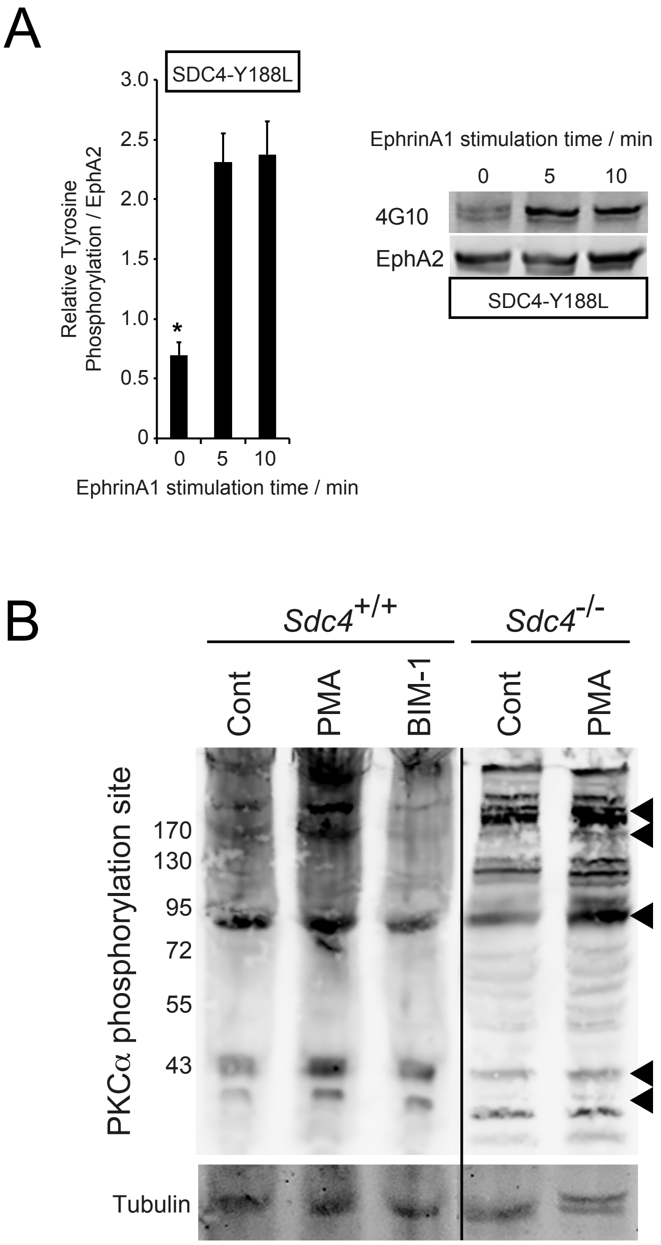


**Supplementary Figure S3. PKCα -mediated effects of SDC4.**

(A) *Sdc4*$^{-/-}$ MEFs stably rescued with SDC4-Y188L were stimulated with 1 µg/ml clustered ephrinA1-Fc before immunoprecipitating EphA2 and blotting for total phosphotyrosine, n=4. Histograms represent phosphorylation divided by EphA2 abundance, determined by quantitative western blot. Error bars represent standard error, significance was tested by ANOVA, * $p<0.05$. (B) *Sdc4*$^{+/+}$ and *Sdc4*$^{-/-}$ MEFs were treated daily with 100 nM PMA or 200 nM BIM-1 for 5 days and lysates blotted with an antibody against serine-phosphorylated PKC substrate consensus motif. n=4.

| Gene | Species | siRNA Sequence | Company |
|---|---|---|---|
| *Sdc4* | *H. sapiens* | CCAAGAAACUAGAGGAGAATT | Silencer® Select, Life Technologies |
| *Sdc4* | *M. musculus* | CGAAGGCAGUUACGACUUG | ON TARGET$^{TM}$ Dharmacon |
| *EphA2* | | GCCGAGCGCAUCUUUAUUG | |
| *Prkca* | | GAAGGGUUCUCGUAUGUCAUU | |
| | | UAACAUAGACCAAUCUGAUUU | |

**Supplementary Table S1. siRNA sequences and sources.**

**Supplementary Video S1. *Sdc4*$^{+/+}$ MEFs exhibit following behaviour after collision**

Related to Figure 1. *Sdc4*$^{+/+}$ MEFs were seeded onto 5-µm fibronectin stripes and time-lapse images were captured every 5 minutes. The video is representative of 29 analysed collisions, *Sdc4*$^{+/+}$ MEFs tended to move in a single direction after collision.

**Supplementary Video S2. *Sdc4*$^{-/-}$ MEFs exhibit repulsion behaviour after collision**

Related to Figure 1. *Sdc4*$^{-/-}$ MEFs were seeded onto 5-µm fibronectin stripes and time-lapse images were captured every 5 minutes. The video is representative of 29 analysed collisions, *Sdc4*$^{-/-}$ MEFs tended to move in opposite directions after collision.

**Supplementary Video S3 *Sdc4*$^{+/+}$ MEFs exhibit a weak contraction response to ephrinA1**

Related to Figure 4. *Sdc4*$^{+/+}$ MEFs were spread on 5µg/ml fibronectin-coated dishes and stimulated with 1µg/ml clustered ephrinA1-Fc. Time-lapse images were captured every 30 seconds for the 10 minutes before and after addition of ephrinA1-Fc (black frames). Time stamps relative to addition of ephrinA1-Fc marked on each frame in seconds. The video is representative of 47 cell responses over 3 experiments.

**Supplementary Video S4 *Sdc4*$^{-/-}$ MEFs transfected with EphA2-targeting siRNA exhibit a weak contraction response to ephrinA1**

Related to Figure 4. *Sdc4*$^{-/-}$ MEFs transfected with EphA2-targeting siRNA were spread on 5µg/ml fibronectin-coated dishes and stimulated with 1 µg/ml clustered ephrinA1-Fc. Time-lapse images were captured every 30 seconds for the 10 minutes before and after addition of ephrinA1-Fc (black frames). Time stamps relative to addition of ephrinA1-Fc marked on each frame in seconds. The video is representative of 47 cell responses over 3 experiments.

**Supplementary Video S5 *Sdc4*$^{-/-}$ MEFs transfected with non-targeting siRNA exhibit a strong contraction response to ephrinA1**

Related to Figure 4. *Sdc4*$^{-/-}$ MEFs transfected with non-targeting siRNA were spread on 5 µg/ml fibronectin-coated dishes and stimulated with 1 µg/ml clustered ephrinA1-Fc. Time-lapse images were captured every 30 seconds for the 10 minutes before and after addition of ephrinA1-Fc (black frames). Time stamps relative to addition of ephrinA1-Fc marked on each frame in seconds. The Video is representative of 47 cell responses over 3 experiments.

**Supplementary Video S6. *Sdc4*$^{+/+}$ MEFs exhibit following behaviour after collision**

Related to Figure 6. *Sdc4*$^{+/+}$ MEFs were seeded onto 5-μm fibronectin stripes and time-lapse images were captured every 5 minutes. The Video is representative of 93 analysed collisions, cells tended to move in a single direction after collision.

**Supplementary Video S7. *Sdc4*$^{-/-}$ MEFs transfected with non-targeting siRNA exhibit repulsion behaviour after collision**

Related to Figure 6. *Sdc4*$^{-/-}$ MEFs transfected with non-targeting siRNA were seeded onto 5-μm fibronectin stripes and time-lapse images were captured every 5 minutes. The video is representative of 78 analysed collisions, cells tended to move in opposite directions after collision.

**Supplementary Video S8. *Sdc4*$^{-/-}$ MEFs transfected with EphA2-targeting siRNA exhibit repulsion behaviour after collision**

Related to Figure 6. *Sdc4*$^{-/-}$ MEFs transfected with EphA2-targeting siRNA were seeded onto 5-μm fibronectin stripes and time-lapse images were captured every 5 minutes. The video is representative of 14 analysed collisions, cells tended to move in opposite directions after collision.

## Supplementary Methods and Materials

### Cell Culture

The generation of immortalised wild-type, *Scd4* -/- and stable syndecan-4-expressing MEFs was described previously (Bass et al., 2007). MEFs were cultured at 33°C in DME, 10% foetal bovine serum, 4.5 g/l glucose, 2 mM L-glutamine and 20 U/ml IFNγ (Sigma). Telomerase-immortalised human fibroblasts were cultured at 37°C in DME, 15% foetal bovine serum, 4.5 g/l glucose, 25 mM HEPES and 2 mM L-glutamine. For RNAi knockdown, 160 pmol of siRNA (Supplementary Table S1) was transfected into a 90% confluent 25-$cm^2$ flask using Dharmafect 2 reagent (Dharmacon). After 24 hours, cells were passaged and cultured for one day before a second transfection to ensure effective knockdown. Cells were passaged 24 hours after the second transfection and used within 2-3 days. Target protein expression in cells transfected with targeting or non-targeting siRNAs was assessed by Western blotting for EphA2 (clone D7, Millipore), PKCα (BD Transduction Labs) and tubulin (DM1A, Sigma), using DyLight680 and DyLight800-conjugated IgGs (ThermoFisher) and imaged with the OdysseySa system (LI-COR Biosciences). For SDC4 overexpression, MEFs were infected with SDC4-encoding virions harvested from AM-12 retroviral packaging cells transfected with pBABEpuro containing the SDC4 cDNA, as described previously (Bass et al., 2007). For inhibitor studies, media was supplemented with appropriate concentrations of PMA or BIM-1 (Cambridge Bioscience), with media changes daily for 5 days.

### qPCR

RNA was extracted from cells or skin biopsies using TRI Reagent (Sigma). After adding chloroform:isoamyl alcohol (Sigma), samples were agitated for 30 seconds and incubated at room temperature for 3 minutes, followed by centrifugation at 14,000 rpm at 4°C for 15 minutes. The RNA-containing aqueous phase was collected, and 10 μg glycogen (Fermentas) along with 500 μl isopropanol were added. The mixture was incubated at room temperature for 10 minutes, then centrifuged at 14,000 rpm at 4°C for 15 minutes. The resulting RNA pellet was washed with 70% ethanol and resuspended in DNase- and RNase-free water at 56°C for 5 minutes.

### Flow Cytometry

Cells were detached using Cell Dissociation Buffer (Sigma), harvested at 300x g, and resuspended in wash buffer (PBS, 1% BSA, 10 mM EDTA). Cells were then stained with APC-conjugated anti-SDC4 (R&D Systems) and fixed with 1% paraformaldehyde. Flow cytometry analysis was performed using a FACSCanto II flow cytometer (Becton Dickinson) and FlowJo software.
